# Determination of the microbial community of traditional Mongolian cheese by using culture‐dependent and independent methods

**DOI:** 10.1002/fsn3.3117

**Published:** 2022-10-26

**Authors:** Liang Guo, Wei‐Liang Xu, Chun‐Dong Li, Fu‐Chao Wang, Yuan‐Sheng Guo, Mei Ya

**Affiliations:** ^1^ Xilingol Vocational College Xilin Gol Institute of Bioengineering Xilinhot China

**Keywords:** microbiota, Mongolian cheese, nutrition, shelf‐life

## Abstract

Mongolian cheese is not only a requisite source of food for the nomadic Mongolian but also follows a unique Mongolian dairy artisanal method of production, possessing high nutritional value and long shelf‐life. In this study, the ancient technique for the production of Mongolian cheese was investigated. The nutritional value of Mongolian cheese was characterized by its high‐protein content (30.13 ± 2.99%) and low‐fat content (9.66 ± 3.36%). *Lactobacillus*, *Lactococcus,* and *Dipodascus* were the predominant bacterial and fungal genera, and *Lactobacillus helveticus, Lactococcus piscium*, and *Dipodascus geotrichum* were the predominant species in the Mongolian cheese. The microbiota of products from different cheese factories varies significantly. The high‐temperature (85°C–90°C) kneading of coagulated curds could eliminate most of the thermosensitive microorganisms for extending the shelf‐life of cheese. The indigenous spore‐forming microbes, which included yeasts, belonging to *Pichia* and *Candida* genera, and molds, belonging to *Mucor* and *Penicillium* genera, which originated from the surroundings during the process of cooling, drying, demolding, and vacuum packaging could survive and cause the package to swell and the cheese to grow mold. The investigation of production technology, nutrition, microbiota, and viable microbes related to shelf‐life contributes to the protection of traditional technologies, extraction of highlights (nutritional profiles and curd scalding) for merchandise marketing, and standardization of Mongolian cheese production, including culture starters and aseptic technique.

## Graphical abstract

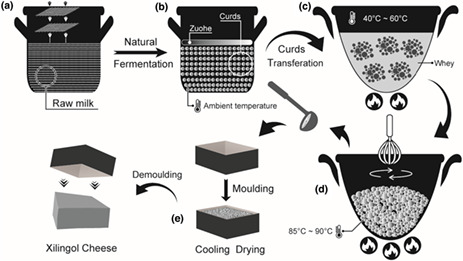

## 1 INTRODUCTION

**FIGURE 1:**
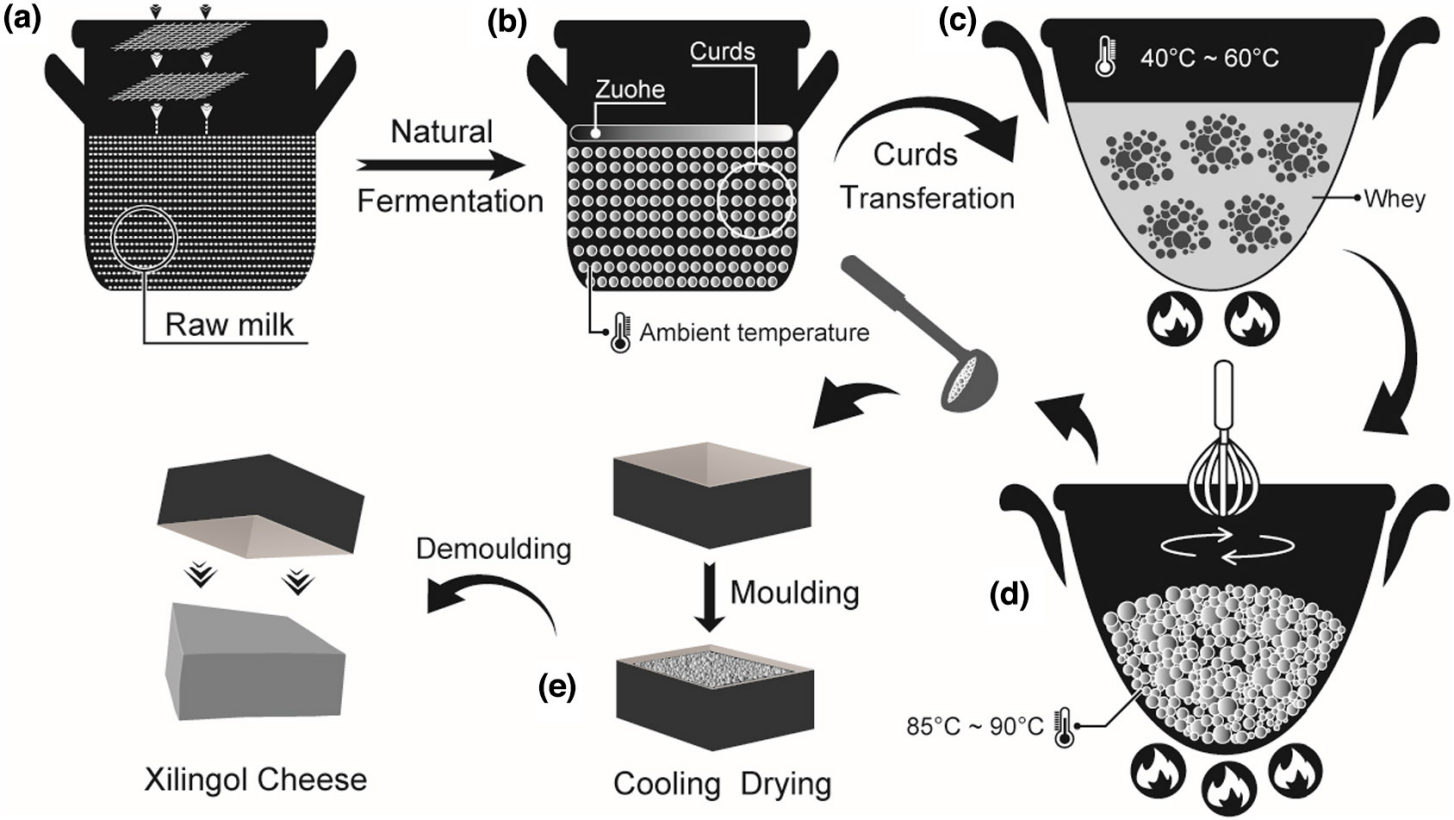
Flow diagram, illustrating the traditional production of Mongolian cheese with quantitative acidity and temperature

Mongolian cheese is known for thousands of years for its culinary history in inner Mongolia, China. From a historical perspective, Mongolian cheese has played a vital role in the nomadic life of Mongolian people and supplies for Mongolian cavalry in wars. Nowadays, Mongolian cheese is the most famous traditional fermented dairy product consumed by the residents of inner Mongolia and is produced by unique traditional craft (Figure 1). Nevertheless, the ancient technique of Mongolian cheese production has rarely been investigated.

Cheese making begins with the natural fermentation of raw milk filtered using multilayered gauze (Figure 1a). The raw milk comes from cows, sheep, and camels. The natural fermentation continues spontaneously at ambient temperature for 1–2 days (Figure 1b). The end of natural fermentation depends on the sensory experience of the maker such as the sour taste of semisolid gels (curds), the appearance of upper sour cream, and the firmness of curd. After removing the upper sour cream (Zuohe), the curd is transferred from the vat to a pot, which is heated using a low flame for separating the coagulated curd followed by draining the whey with the help of scoop (Figure 1c). The coagulated curd is then heated using a high flame and kneaded into a high‐fluid state followed by the removal of whey (Figure 1d). The fluid cheese is molded into a wooden box, which is then cooled and dried (Figure 1e). The abundant field investigation for the production of cheese showed that the temperature of natural fermentation (18–20°C) was lower than the average room temperature, where the fermentation vat was kept in a cool closet in dark; the natural fermentation may reach 75°T (acidity) and pH 4.0 at the end. In addition, the curd is heated to 40–60°C for removing the whey, while the coagulated curd is heated to 85–90°C and kneaded continuously for producing a smooth and fluid cheese. The ambient temperature ensures the continuation of natural fermentation till the end and endows a distinct flavor into Mongolian cheese by increasing flavor‐producing substances. The duration of fermentation depends on the microbiota of raw milk, sanitary conditions of vat and atmosphere, and ambient temperature. This artisanal production process of Mongolian cheese, including acidity and temperature of fermentation, draining of whey, and kneading of coagulated curd contributes to the standardized production of Mongolian cheese.

The traditional production of Mongolian cheese and the unique environmental conditions of the Mongolian plateau endow it with characteristic nutrition and microbiota. Although various traditional cheeses have been investigated worldwide for their production method (Aboudoulaye & Kaya, 2020; Cuevas‐Gonzalez et al., 2017; Johler et al., 2018; Nogueira Silva et al., 2020), physicochemical characteristics (Cuevas‐Gonzalez et al., 2017; Ercan et al., 2011; Nogueira Silva et al., 2020; Sanchez‐Macias et al., 2010; Sulejmani et al., 2014), shelf lives (Al‐Moghazy et al., 2020; Alwazeer et al., 2020; Atallah et al., 2021; Masotti et al., 2012; Ramos et al., 2012; Tayebi‐Moghaddam et al., 2021), functional strains (Bian et al., 2016; Cui et al., 2018; Muhammad et al., 2019; Saleem et al., 2021; Shi et al., 2019), and microbiota (Biolcati et al., 2020; Jin et al., 2018; Jingkai et al., 2020; Nam et al., 2021; Yunita & Dodd, 2018; Zhang et al., 2021), the studies related to Mongolian cheese are limited. These studies only focused on the antimicrobial, antioxidant, and probiotic potential of *Lactobacillus plantarum* strains isolated from Mongolian cheese (Min et al., 2019; Muhammad et al., 2019; Zhang et al., 2014) and the storage (Mei et al., 2013; Wei et al., 2021) and microbial diversity (Liang et al., 2021; Yang et al., 2020, 2021) of Mongolian cheese. As the studies on Mongolian cheese might increase and win universal praise, the fundamental investigations, including artisanal production method, nutrition, and microbiota are needed to be carried out. In the present study, the traditional production of Mongolian cheese was investigated in field practices and its quantification indicators and nutritional contents were measured using a large number of samples followed by the identification of microbial composition, including bacteria and fungi. Last, the viable microbes in the Mongolian cheese were quantified, isolated, and identified to investigate the underlying reasons, affecting its shelf life.

## 2 MATERIALS AND METHODS

### 2.1 A sampling of artisanal Mongolian cheese

**FIGURE 2:**
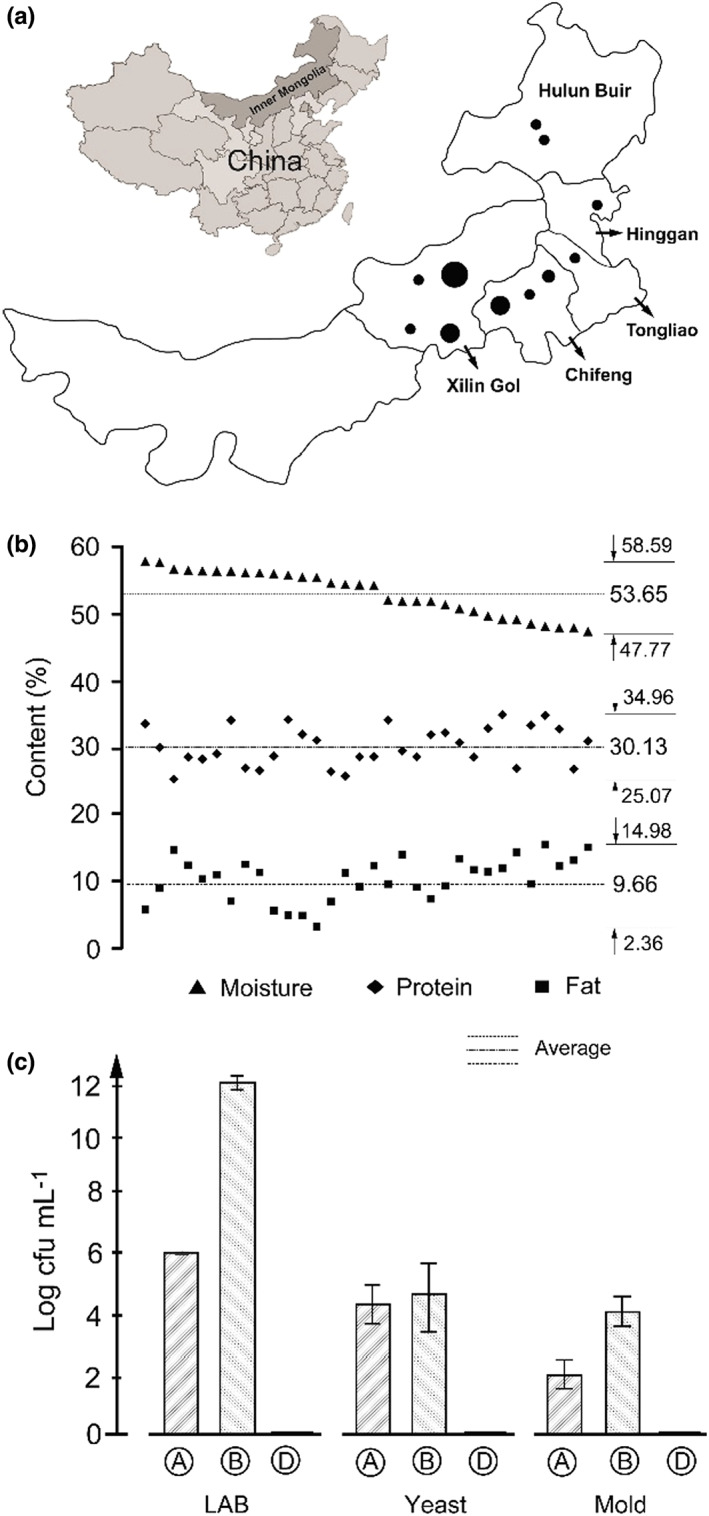
Physicochemical composition and viable microbial dynamics during the production of Mongolian cheese. Sampling of Mongolian cheese in the five administrative divisions of inner Mongolia and its geographical position in China is highlighted (a). Contents of moisture, protein, and fat were measured as per the national food safety standards in China (b). Dynamics of LAB, yeast, and mold counts were demonstrated via histogram during the production of Mongolian cheese at three time points (Figure 1a,b,d) from three cheese factories (c)

A total of 73 artisanal Mongolian cheese samples were collected from Mongolian nomads in the major cheese‐producing areas, including Xilin Gol, Chifeng, Tongliao, Higngan, and Hulun Buir in inner Mongolia (Figure 2a). A total of 11, 1, 1, and 6 samples were collected from Xilinhot, Abag Banner, Huang Banner, and Lan Banner in Xilin Gol, while 9, 1, 1, and 2 samples were collected from Chifeng, Higngan, Hulun Buir, and Tongliao (Figure 2a). These cheese samples (1 kg) were loaded promptly into the sterile self‐sealing bags and stored at −80°C for nutritional and microbiological analysis by using culture‐dependent methods. A total of 27 artisanal Mongolian cheese samples were obtained from the three artisanal cheese factories (F, Fenghua; S, Suainiu; and M, Muxiangyuan) for microbiota analysis, and nine samples were collected from each factory and three parallel samples were obtained from the same period of production by the same factory.

### 2.2 Measurement of moisture, protein, and fat contents and abundance of viable microorganisms

The contents of moisture (China National Food Safety Standard, 2016a), protein (China National Food Safety Standard, 2016b), and fat (China National Food Safety Standard, 2016c) were measured as per national food safety standards in China. The protein and fat contents were measured using the Kjeldahl method and Soxhlet extraction method, respectively. The abundance of total bacterial count (TBC) and lactic acid bacteria (LAB) was measured for 24 h at 37°C using nutrient agar medium (China National Food Safety Standard, 2016d) and 72 h at 36°C using Man Rogosa Sharp (MRS) medium (China National Food Safety Standard, 2016e), while the yeast and mold were calculated for 5 days at 28°C using Rose Bengal agar medium (China National Food Safety Standard, 2016f).

### 2.3 16 S rRNA and ITS gene sequencing, bioinformatics, and statistical analysis

The total microbial DNA was extracted and purified using E.Z.N.A stool DNA kit (Omega Bio‐tek, Norcross, US). The V3‐V4 hypervariable region of *16 S rRNA* gene was amplified with 341F 5′‐CCTACGGGNGGCWGCAG‐3′ and 806R 5′‐GGACTACHVGGGTATCTAAT‐3′ primers. The *ITS* gene sequence was amplified with ITS3‐KYO2F 5′‐GATGAAGAACGYAGYRAA‐3′ and ITS4R 5′‐TCCTCCGCTTATTGATATGC‐3′. The PCR reaction mixture consisted of the following: 5 μl of 10 X KOD buffer, 1 μl of KOD polymerase, 5 μl of 2.5 mM dNTPs, 1.5 μl of each primer (5 μM), and 100 ng of microbial DNA. The thermal program of PCR was as follows: initial denaturation at 95°C for 2 min; followed by 27 cycles of denaturation at 98°C for 10 s, annealing at 62°C for 30 s, and extension at 68°C for 30 s; and final extension at 68 °C for 10 min. The amplicons were quantified and subjected to paired‐end sequencing (2 × 250) using the Illumina MiSeq platform (Illumina, San Diego, CA). The high‐quality sequencing reads were acquired by removing the reads with more than 10% of unknown nucleotides and those with less than 80% of bases with quality‐value (Q‐value) >20. The final effective reads were obtained by trimming the chimeric tags and were clustered into the operational taxonomic units (OTUs) with ≥97% similarity using the UPARSE pipeline (Edgar, 2013). The OTUs were classified into organisms with the Naive Bayesian Model using RDP classifier (Wang et al., 2007) based on SILVA database for *16 S rRNA* gene sequencing (Pruesse et al., 2007) and UNITE database for *ITS* gene sequencing (Koljalg et al., 2005).

## 3 RESULTS AND DISCUSSION

### 3.1 Nutritional and microbiological investigation of Mongolian cheese

There are approximately 18 types of cheese varieties based on the classification scheme of production methods, including curding, cutting the coagulum, stirring, draining, heating, pressing, salting, and ripening (Walter & Hargrove, 1972). In general, Mongolian cheese is classified as an acidic curd cheese for the natural fermentation of raw milk to 75°T (acidity) and pH 4.0 (Figure 1). The acid‐coagulated Mongolian cheese is characterized by high moisture (53.65 ± 3.46%) and protein contents (30.13 ± 2.99%) and low‐fat content (9.66 ± 3.36%) for cream floating during natural fermentation (Figure 1b). The superficial floating cream in coagulum is named Zuohe in Mongolian. In order to fully understand the conventional nutritional potential of Mongolian cheese, a total of 32 samples were collected from the Mongolian nomads in the major cheese‐producing areas including Xilin Gol, Chifeng, Tongliao, Hinggan, and Hulun Buir (Figure 2a). The moisture content ranged from 47.77% to 58.59%, and the protein and fat contents ranged from 25.07% to 34.96% and from 2.36% to 14.98%, respectively (Figure 2b). The Mongolian nomads heat coagulum and stir it to a high‐fluid state based on their experience and preference and usually consume or sell it soon after production. The intensity of removing whey, stirring, and drying was slightly different from the artisanal production methods by Mongolian nomads and determined the moisture content within limits. The changes in acidity during natural fermentation could influence the degree of protein coagulation and cream floating, thereby affecting the corresponding contents.

The changes in microbial abundance in the manufacturing process of Mongolian cheese were analyzed by sampling at three time points (Figure 1a, b) from three cheese factories. The total LAB count ranged from 1.17 × 10^6^, 9.65 × 10^5^, and 1.29 × 10^6^ cfu/g in the raw milk (Figure 1a) to 1.31 × 10^12^, 3.41 × 10^12^, and 1.28 × 10^12^ cfu/g at the end of fermentation, respectively (Figure 1b), while the LAB count was 0 cfu/g in the Mongolian cheese that had just been produced (Figure 1). The yeast count ranged from 6.45 × 10^4^, 1.79 × 10^4^, and 7.3 × 10^3^ cfu/g in the raw milk to 5.9 × 10^5^, 1.5 × 10^4^, and 1.1 × 10^4^ cfu/g at the end of fermentation, respectively, while the yeast count was 0 cfu/g in Mongolian cheese. Similarly, the mold count ranged from 100, 50, and 2.25 × 10^3^ cfu/g in the raw milk to 5 × 10^3^, 4.9 × 10^4^, and 1.15 × 10^4^ cfu/g at the end of fermentation, while the mold count was 0 cfu/g in Mongolian cheese that had just been produced. As shown in Figure 2c, the enrichment of microbiota, including LAB, yeast, and mold in the natural fermentation process promoted the milk to form curd and endow Mongolian cheese with fermentative flavor. The coagulated curd was then heated to 85–90°C for producing a smooth and fluid cheese and eliminating most of the thermosensitive microorganisms to increase its shelf‐life.

### 3.2 Abundance and composition of microbiota in Mongolian cheese

Although the Mongolian cheese, which had just been produced, did not have viable LAB, yeast, and mold, these microorganisms play a vital role in the formation of coagulated curd and flavor of cheese. The composition of the microbiota, including bacteria and fungi, which existed during the production of cheese were investigated using *16 S rRNA* and *ITS* genes sequencing technology.

After removing the low‐quality and chimeric reads, a total of 483,286, 407,725, and 459,731 bacterial reads (Average ± SD: 53,698 ± 7389; 45,303 ± 3926; 51,081 ± 5701) and 645,354, 633,337, and 472,240 fungal reads (Average ± SD: 71,706 ± 1833; 70,371 ± 2243; 52,471 ± 6221) were obtained from the three artisanal cheese factories (F, Fenghua; S, Suainiu; and M, Muxiangyuan), and nine samples were collected from each factory. The OTUs, Chao1, Shannon, Simpson, and Good's coverage indices were used to evaluate the enrichment and diversity of microbial communities. The Shannon curves, but not the rarefaction curves, reached saturation in the sequencing of *16 S rRNA* and *ITS* genes (Table S1 and Figure S2). This indicated that the sequencing depth was sufficient to represent the whole bacterial and fungal communities. There were significant differences in the diversity of bacterial and fungal communities among Mongolian cheese from the three artisanal factories (*p* < 0.001), while the microbial diversity did not alter during the three different periods of production by the same manufacturer (*p* > 0.05). The different sources of raw milk and production atmospheres showed that the bacterial and fungal communities were involved in natural fermentation, thereby ultimately affecting the coagulation.

**FIGURE 3:**
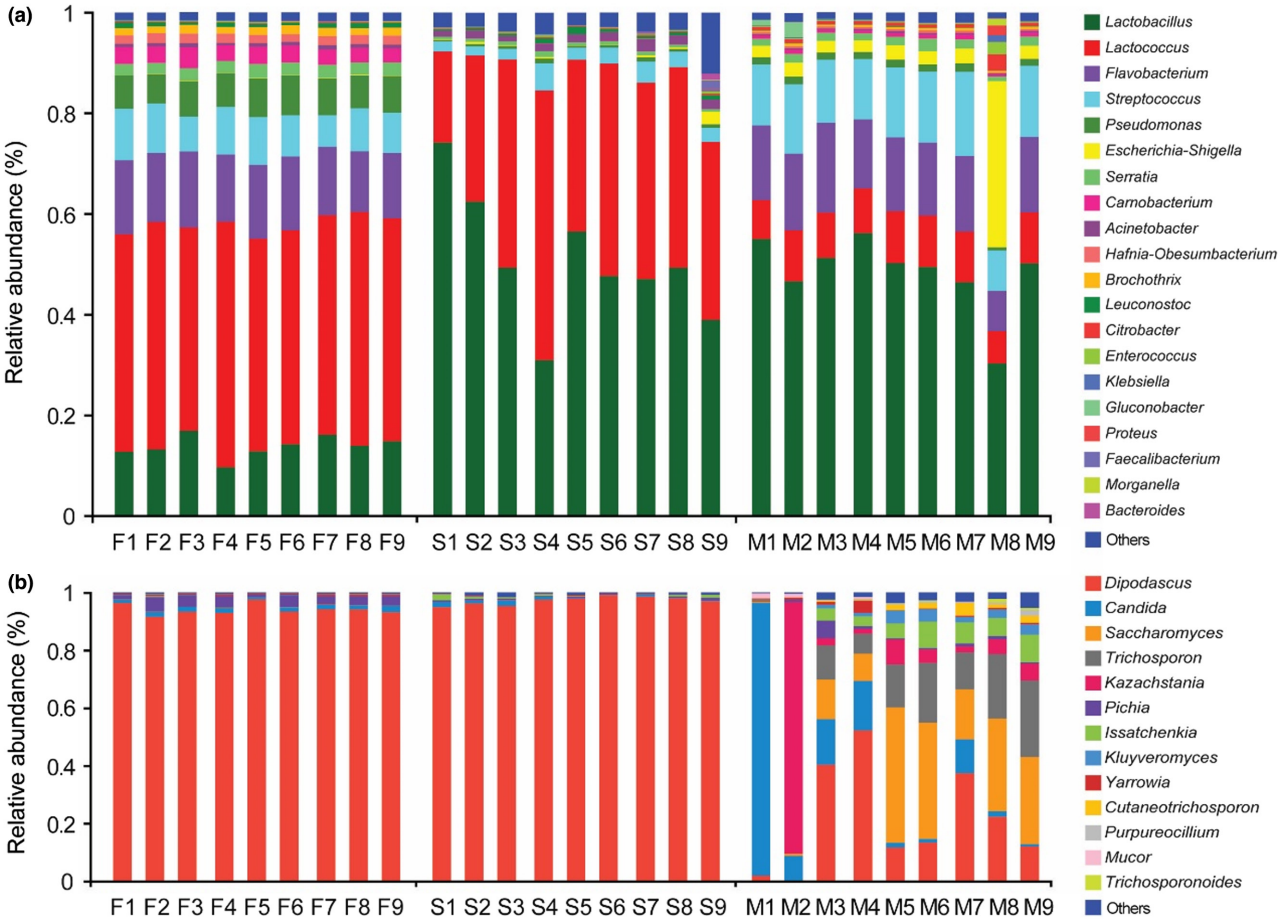
Relative abundance of bacterial (a) and fungal (b) sequences at genus level in the nine individual samples from three artisanal cheese factories (F: Fenghua; S: Suainiu; M: Muxiangyuan)

Analyzing the abundance of bacterial and fungal communities as phylum level showed that the phyla Firmicutes, Bacteroidetes, Proteobacteria, and Actinobacteria represented 73.53 ± 1.49%, 13.13 ± 0.96%, 13.31 ± 0.90%, and 0.03 ± 0.01% in the Fenghua, 93.10 ± 2.27%, 0.84 ± 0.59%, 5.47 ± 1.53%, and 0.59 ± 0.35% in the Suainiu, and 72.41 ± 8.89%, 14.25 ± 2.49%, 13.23 ± 11.10%, and 0.11 ± 0.04% in the Muxiangyuan samples, respectively, while the phyla Ascomycota and Basidiomycota represented 99.89 ± 0.05% and 0.11 ± 0.05% in the Fenghua, 99.88 ± 0.29% and 0.11 ± 0.29% in the Suainiu, and 84.57 ± 10.18% and 14.93 ± 10.73% in the Muxiangyuan samples. As shown in Figure 3a; nine bacterial genera (>1%) were identified in the Fenghua samples, including *Lactococcus* (41.65%), *Flavobacterium* (12.98%), *Lactobacillus* (13.04%), *Streptococcus* (8.03%), *Pseudomonas* (6.58%), *Carnobacterium* (3.07%), *Serratia* (2.32%), *Hafnia‐Obesumbacterium* (1.67%), and *Brochothrix* (1.47%); three bacterial genera (>1%) were identified in the Suainiu samples, including *Lactobacillus* (49.69%), *Lactococcus* (37.18%), and *Streptococcus* (3.04%); and seven bacterial genera (>1%) were identified in the Muxiangyuan samples, including *Lactobacillus* (46.47%), *Flavobacterium* (13.68%), *Streptococcus* (12.53%), *Lactococcus* (8.87%), *Escherichia‐Shigella* (6.42%), *Serratia* (1.58%), and *Pseudomonas* (1.32%). As shown in Figure 3b; three fungal genera (>1%) were identified in the Fenghua samples, including *Dipodascus* (94.03%), *Pichia* (3.16%), and *Candida* (1.53%); only one fungal genus (*Dipodascus* 97.13%) was identified in the Suainiu samples; and nine fungal genera (>1%) were identified in the Muxiangyuan samples, including *Dipodascus* (22.46%), *Saccharomyces* (21.03%), *Candida* (18.46%), *Trichosporon* (12.87%), *Kazachstania* (10.61%), *Issatchenkia* (5.06%), *Kluyveromyces* (2.17%), *Cutaneotrichosporon* (1.39%), and *Pichia* (1.37%).

The abundance of bacteria at species level showed that nine bacterial species (>1%) were identified in the Fenghua samples, including *Lactococcus piscium* (20.75%), *Flavobacterium frigidarium* (11.99%), *Lactobacillus helveticus* (12.54%), *Streptococcus salivarius* (7.18%), *Pseudomonas fragi* (5.52%), *Carnobacterium maltaromaticum* (3.07%), *Rahnella aquatilis* (2.05%), *Hafnia paralvei* (1.67%), and *Brochothrix thermosphacta* (1.47%); four bacterial species (>1%) were identified in the Suainiu samples, including *Lactobacillus helveticus* (46.32%), *Lactococcus piscium* (8.08%), *Lactobacillus reuteri* (2.91%), and *Streptococcus salivarius* (1.77%); and nine bacterial species (>1%) were identified in the Muxiangyuan samples, including *Lactobacillus helveticus* (45.09%), *Flavobacterium frigidarium* (13.53%), *Streptococcus lutetiensis* (7.34%), *Escherichia coli* (6.40%), *Lactococcus piscium* (5.67%), *Streptococcus agalactiae* (2.15%), *Rahnella aquatilis* (1.53%), *Streptococcus salivarius* (1.51%), and *Streptococcus parauberis* (1.42%).

The abundance of fungi at species level showed that three fungal species (>1%) were identified in the Fenghua samples, including *Dipodascus geotrichum* (94.03%), *Pichia fermentans* (3.02%), and *Candida sake* (1.26%); only one fungal species *Dipodascus geotrichum* (97.13%) (>1%) was identified in the Suainiu samples; and six fungal species (>1%) were identified in the Muxiangyuan samples, including *Dipodascus geotrichum* (22.46%), *Candida zeylanoides* (17.80%), *Trichosporon asteroids* (12.87%), *Issatchenkia orientalis* (5.06%), *Kazachstania unispora* (2.82%), and *Kluyveromyces marxianus* (2.14%).

**FIGURE 4:**
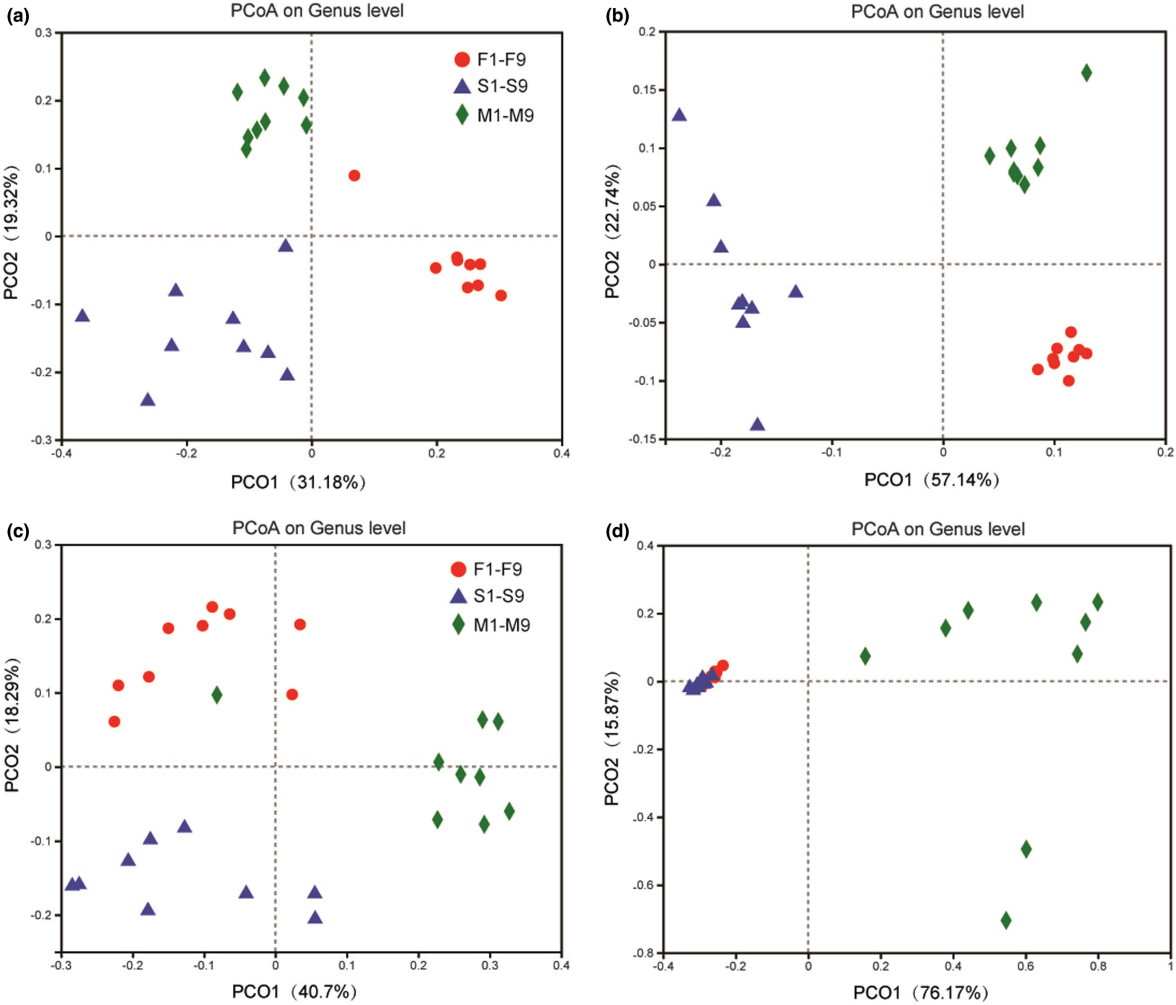
Unweighted (a,c) and weighted (b,d) UniFrac principal coordinate analyses of the bacterial (a,b) and fungal (c,d) diversities in the nine individual samples from three artisanal cheese factories (F: Fenghua; S: Suainiu; M: Muxiangyuan)

Multivariate analysis was performed to compare the compositions of bacterial and fungal communities among the three artisanal cheese factories. Figure 4a (for unweighted PCoA) and Figure 4b (for weighted PCoA), which used genus‐level OTUs in *16 S rRNA* gene sequencing, show significant differences in the bacterial communities among the samples from three artisanal cheese factories (ANOSIM: R = 0.711, *p* = 0.001 for unweighted PCoA; R = 0.891, *p* = 0.001 for weighted PCoA). Similarly, Figure 4c (for unweighted PCoA) and Figure 4d (for weighted PCoA), which used genus‐level OTUs in *ITS* sequencing, show significant differences in the fungal communities among the samples from three artisanal cheese factories (ANOSIM: R = 0.766, *p* = 0.001 for unweighted PCoA; R = 0.609, *p* = 0.001 for weighted PCoA).

### 3.3 Quantification, isolation, and identification of viable microbes from Mongolian cheese

**FIGURE 5:**
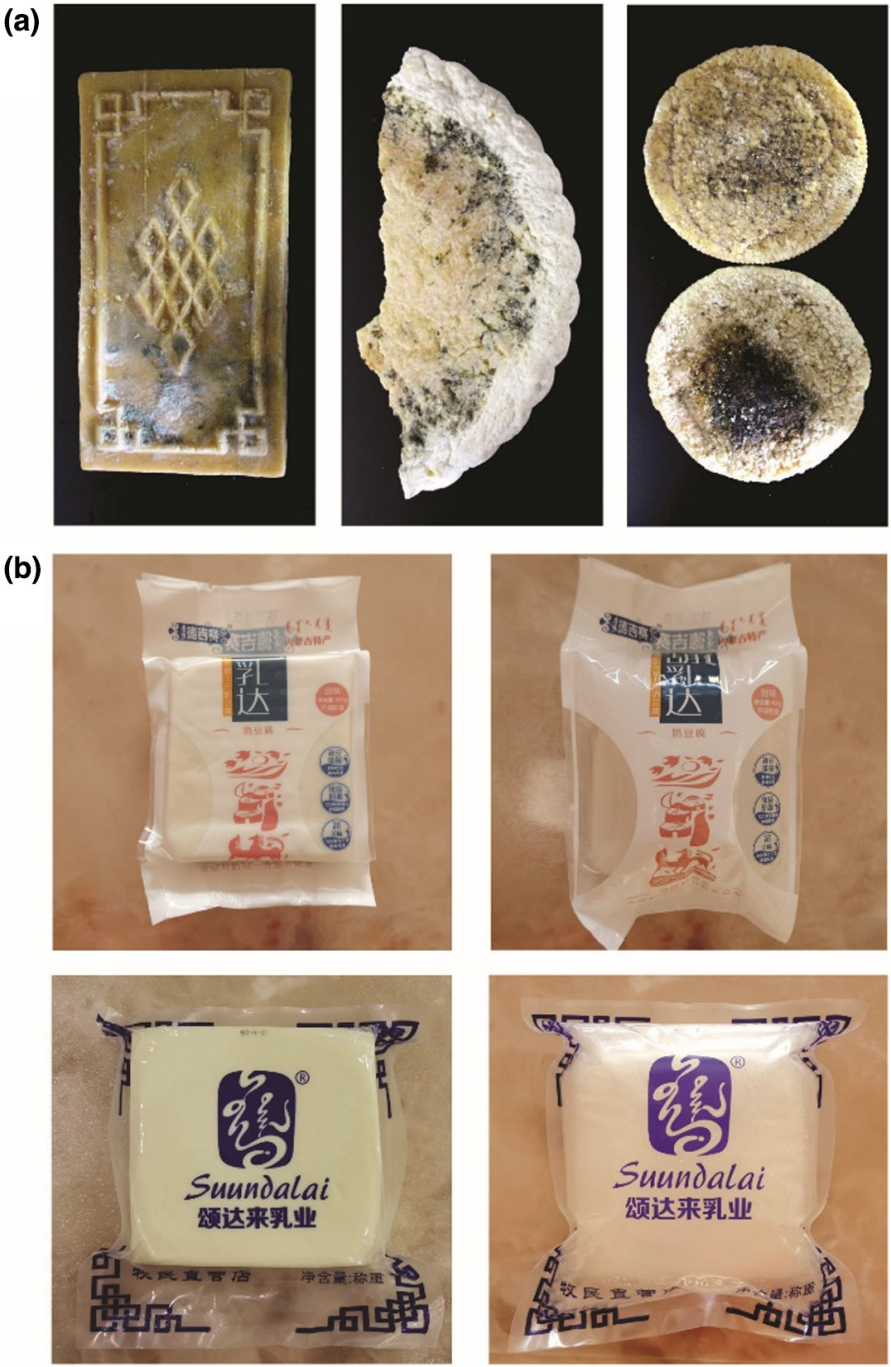
Moldy Mongolian cheese (a) and plastic package ballooning (b)

**FIGURE 6:**
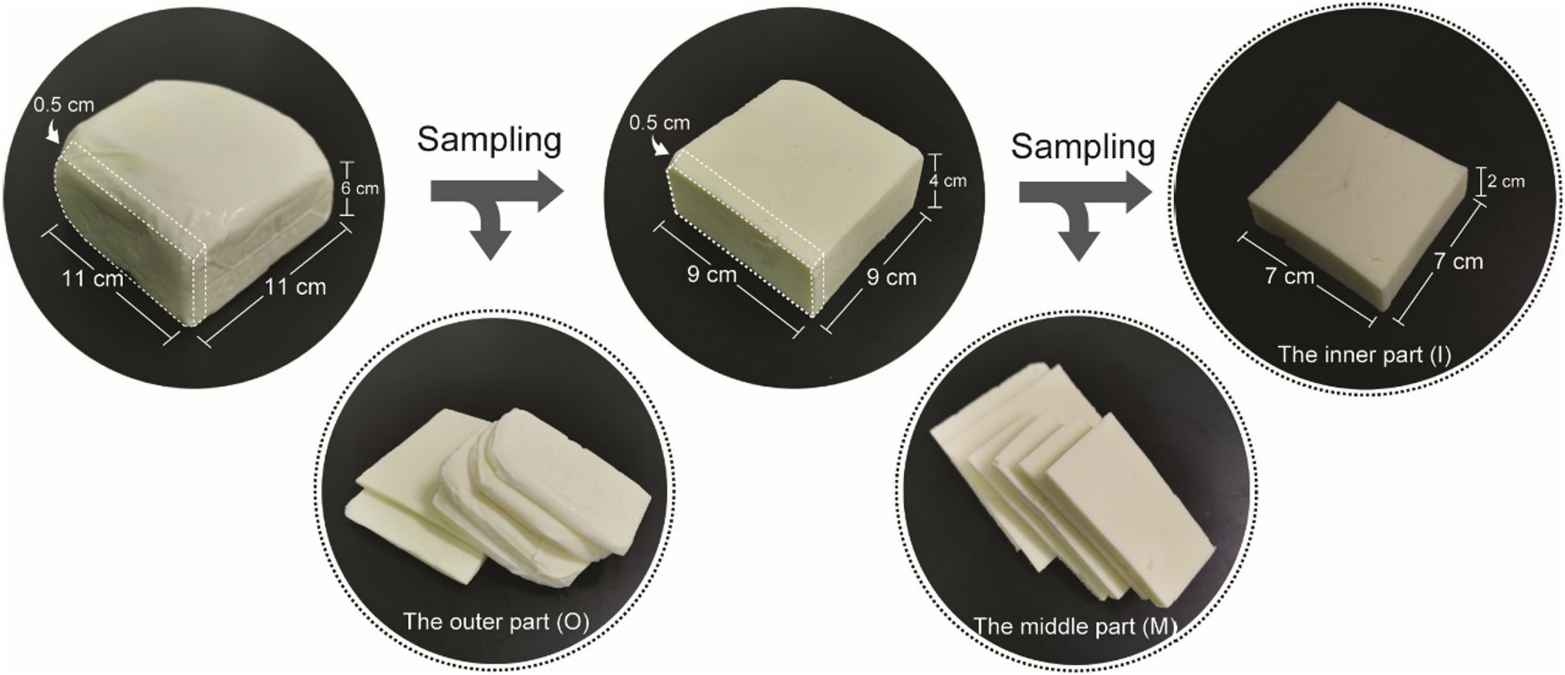
Cheese was divided into three parts (the outer part; the middle part; the inner part) from the outside to the inside

**FIGURE 7:**
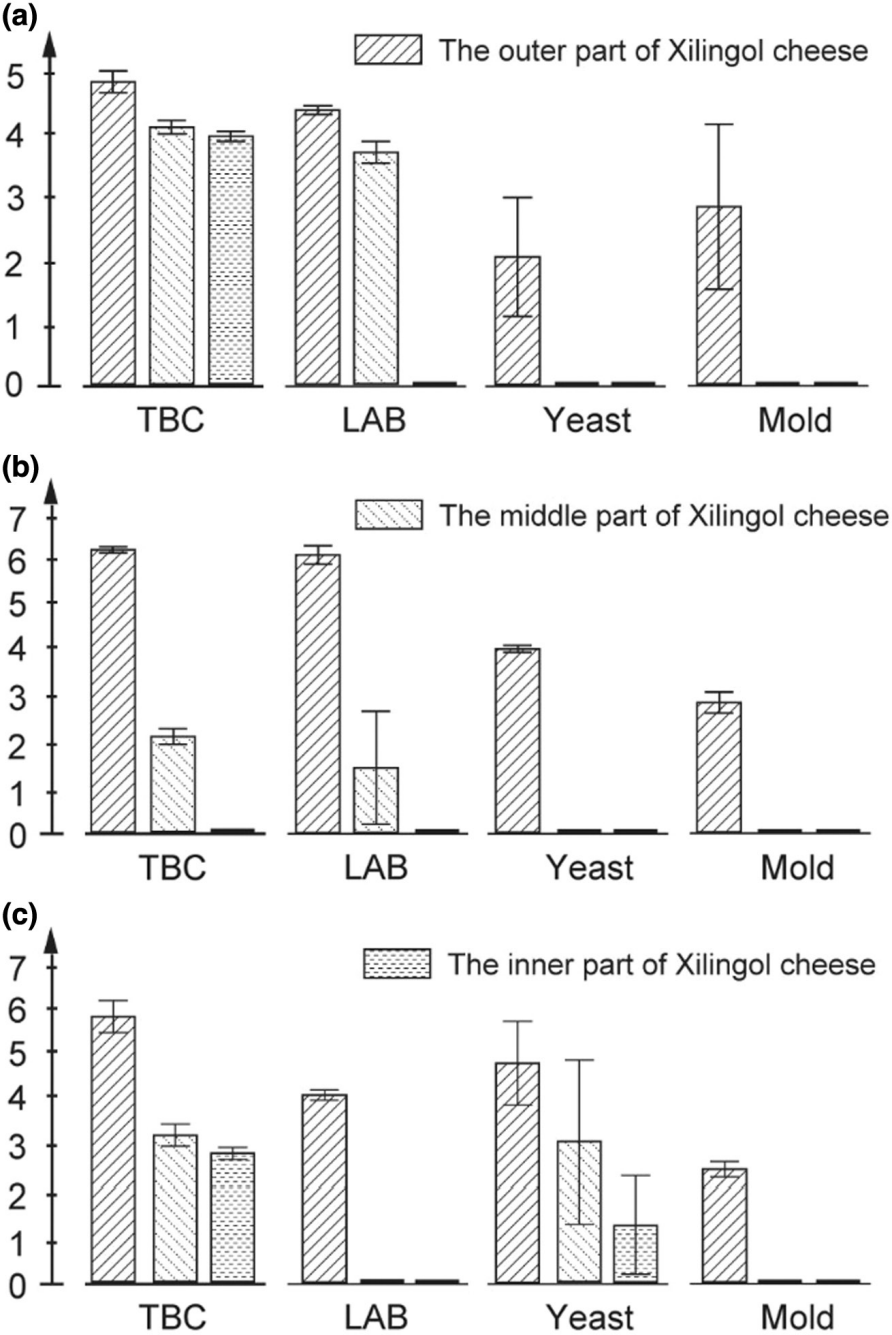
The TBC, LAB, yeast, and mold counts were determined using culture‐dependent methods in the samples from the outer part, the middle part, and the inner part of cheese (a: Fenghua; b: Suainiu; c: Muxiangyuan)

**FIGURE 8:**
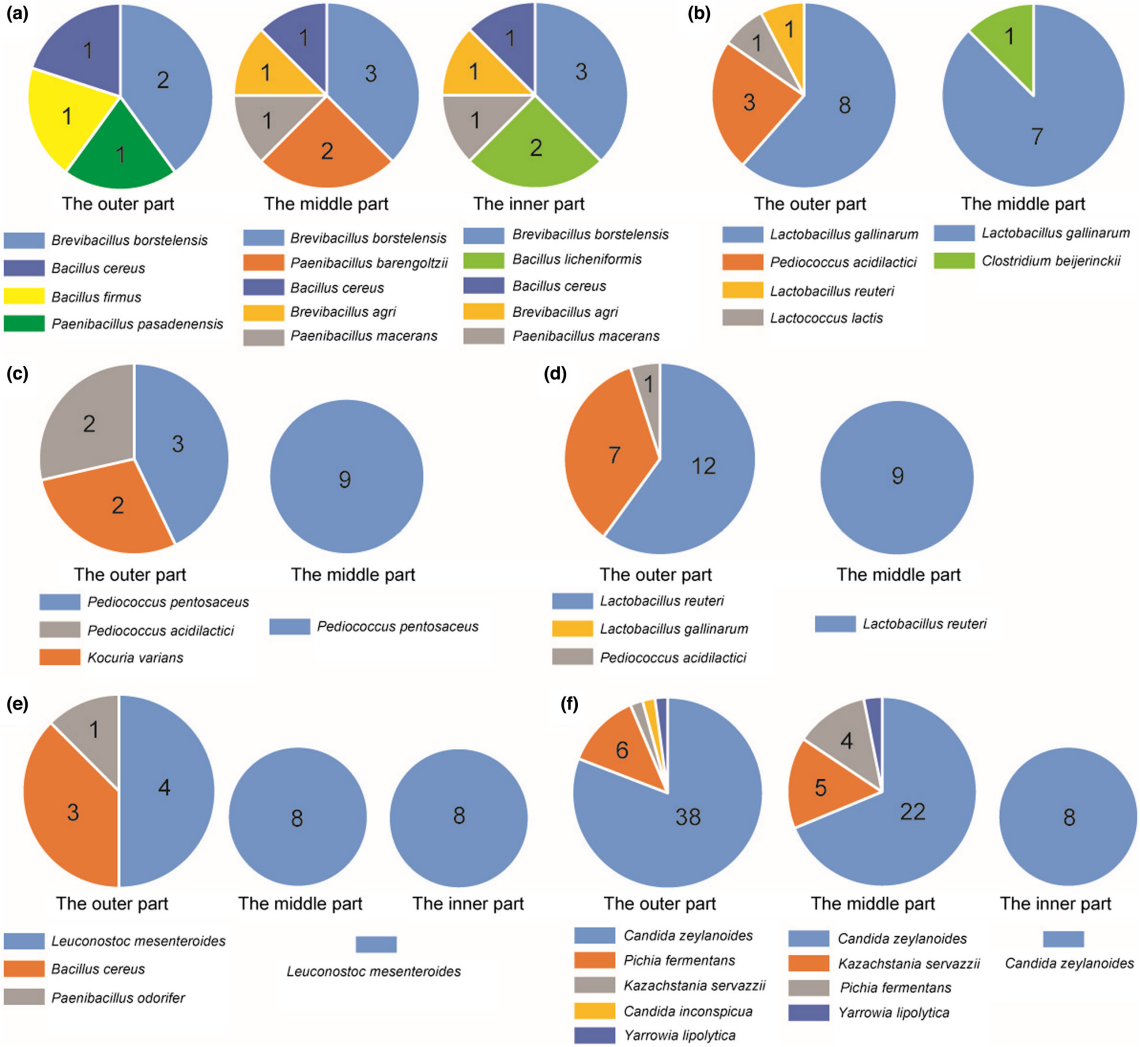
The species diversity and abundance of TBC, LAB and yeast were determined using ITS sequencing method in the samples from the outer part, the middle part and the inner part of cheese (a and b: fenghua; c and d: Suainiu; e and f: Muxiangyuan)

A great diversity of bacterial and fungal species were discovered in the Mongolian cheese using amplicon sequencing. The microbiota in Mongolian cheese contained all the microorganisms residing in raw milk as well as those surrounding the environment of production, which were eliminated using high temperatures (85–90°C) (Figure 1d and Figure 2c). Nevertheless, cheese could easily get mildewed and its wrapping was liable to swell up (Figure 5). It was speculated that the viable microbes, which affect the shelf life of cheese, might originate from the surroundings during the process of cooling, drying, demolding, and wrapping. In order to verify this hypothesis, the artisanal cheese from the three different factories was divided into three parts (the outer part; the middle part; the inner part) from the outside to the inside (Figure 6). The TBC, LAB, yeast, and mold counts were determined using culture‐dependent methods in the samples from the three parts. Figure 7a shows that the TBC and LAB counts in the outer part of cheese from Fenghua were significantly more as compared to those in the middle and inner parts (*p* < 0.05), while the yeast and mold did not exist in the middle and inner parts. The species diversity and abundance of TBC did not change from the outer to the inner layer of cheese (Figure 8a), while the diversity of LAB decreased from the outer to the middle layer of cheese (Figure 8b). As shown in Figure 7b, the TBC and LAB count in the outer part of cheese from Suainiu was significantly more as compared to those in the middle part (*p* < 0.05), while yeast and mold did not exist in the middle and inner parts. The diversity of TBA and LAB decreased from the outer to the middle layer of cheese (Figure 8c,d). As shown in Figure 7, the TBC and yeast counts in the outer part of cheese from Muxiangyuan were significantly more than those in the middle and inner parts (*p* < 0.05), while LAB and mold did not exist in the middle and inner parts. The diversity of TBC and yeast decreased from the outer to the middle layer of cheese (Figure 8e,f). In conclusion, the indigenous bacteria and fungi, which existed in the environment of production, including cooling, drying, demolding, and wrapping, colonized the surface of cheese, causing the swelling of packages and the formation of mold. The yeast species, belonging to *Pichia* and *Candida*, can produce carbon dioxide, and the molds, belonging to *Mucor* and *Penicillium*, grow on the surface of cheese. Some spore‐forming molds also overcome high temperatures (85–90°C) and survive, affecting the shelf life of cheese.

## 4 CONCLUSIONS

Mongolian cheese has been a cultural icon of food since the ancient days. In this study, the scientific implications of the ancient technique for the production of Mongolian cheese were explored. The contents of protein and fat were 30.13 ± 2.99% and 9.66 ± 3.36%, respectively. The high‐temperature (85–90°C) kneading of coagulated curds could eliminate most of the thermosensitive microorganisms in cheese. The predominant bacterial and fungal genera included *Lactobacillus*, *Lactococcus,* and *Dipodascus*, and *Lactobacillus helveticus*, *Lactococcus piscium, and Dipodascus geotrichum* were predominant species in the Mongolian cheese. The vacuum‐sealed plastic packs of Mongolian cheese are liable to mildew and swell. Using the quantification, isolation, and identification of microbes in the different spatial positions of cheese, it was found that the indigenous spore‐forming microbes, which survived at high temperature (85–90°C) and included yeasts, belonging to *Pichia* and *Candida,* and molds, belonging to *Mucor* and *Penicillium*, originated from the surroundings during the process of cooling, drying, demolding, and wrapping and might cause the vacuum packages to swell and make the cheese to easily get mildew. According to the experimental results, it was assumed that sterilizing the surroundings of cooling, drying, demolding, and packaging, use of antibacterial packaging materials, and heat sterilization after vacuum packaging might eliminate the microbes on the surface of cheese to some extent, thus increasing its shelf‐life.

## CONFLICT OF INTEREST

All authors declare no conflict of interest.

## ETHICAL APPROVAL

This study does not involve any human or animal testing.

## Supporting information


Table S1
Figure S2

## Data Availability

The data that support the findings of this study are available on request from the corresponding author.
