# Supplementary material for: Determination of the microbial community of traditional Mongolian cheese by using culture‐dependent and independent methods
Source: Food Sci Nutr. 2022 Oct 26;11(2):828–37. doi: 10.1002/fsn3.3117 (PMC9922113; doi:10.1002/fsn3.3117)
Supplement: Supplementary file 1 — Table S1 Figure S2 [file FSN3-11-828-s001.docx]

Supplemental Table S1. Diversity indices of amplicon sequencing of Mongolian cheese in China

|  | Sample | Reads | OTUs | Chao | Shannon | Simpson | Good's coverage |
| --- | --- | --- | --- | --- | --- | --- | --- |
| 16s | F1 | 42567 | 117 | 140 | 2.85 | 0.10 | 0.99940 |
|  | F2 | 48237 | 123 | 136 | 2.79 | 0.11 | 0.99932 |
|  | F3 | 56043 | 121 | 147 | 1.54 | 0.37 | 0.99962 |
|  | F4 | 54967 | 126 | 197 | 2.25 | 0.25 | 0.99931 |
|  | F5 | 52726 | 126 | 173 | 2.81 | 0.11 | 0.99945 |
|  | F6 | 68810 | 148 | 279 | 2.43 | 0.23 | 0.99939 |
|  | F7 | 47766 | 121 | 207 | 1.98 | 0.25 | 0.99951 |
|  | F8 | 56029 | 135 | 179 | 1.63 | 0.33 | 0.99930 |
|  | F9 | 56141 | 128 | 193 | 2.14 | 0.26 | 0.99938 |
|  | S1 | 42295 | 123 | 153 | 1.61 | 0.37 | 0.99933 |
|  | S2 | 53565 | 199 | 203 | 2.01 | 0.31 | 0.99957 |
|  | S3 | 45923 | 214 | 229 | 2.22 | 0.25 | 0.99929 |
|  | S4 | 43965 | 141 | 228 | 1.81 | 0.30 | 0.99950 |
|  | S5 | 41972 | 139 | 171 | 1.72 | 0.31 | 0.99919 |
|  | S6 | 45986 | 166 | 224 | 2.26 | 0.23 | 0.99922 |
|  | S7 | 40191 | 150 | 188 | 2.79 | 0.11 | 0.99922 |
|  | S8 | 47596 | 163 | 143 | 2.85 | 0.10 | 0.99933 |
|  | S9 | 46232 | 272 | 197 | 1.66 | 0.33 | 0.99947 |
|  | M1 | 44562 | 168 | 177 | 1.53 | 0.38 | 0.99886 |
|  | M2 | 57483 | 165 | 140 | 2.78 | 0.11 | 0.99917 |
|  | M3 | 50331 | 186 | 214 | 2.29 | 0.23 | 0.99906 |
|  | M4 | 45650 | 169 | 225 | 2.03 | 0.30 | 0.99898 |
|  | M5 | 50507 | 180 | 182 | 2.81 | 0.11 | 0.99897 |
|  | M6 | 62473 | 172 | 215 | 2.19 | 0.26 | 0.99918 |
|  | M7 | 47228 | 152 | 198 | 2.82 | 0.11 | 0.99907 |
|  | M8 | 49947 | 171 | 219 | 2.83 | 0.11 | 0.99913 |
|  | M9 | 51550 | 179 | 219 | 2.32 | 0.20 | 0.99904 |
|  |  |  |  |  |  |  |  |
| ITS | F1 | 72527 | 37 | 38 | 0.24 | 0.93 | 0.99996 |
|  | F2 | 69863 | 47 | 51 | 0.39 | 0.86 | 0.99990 |
|  | F3 | 72996 | 46 | 55 | 0.33 | 0.89 | 0.99986 |
|  | F4 | 71862 | 49 | 54 | 0.45 | 0.84 | 0.99989 |
|  | F5 | 69396 | 32 | 35 | 0.17 | 0.95 | 0.99993 |
|  | F6 | 73922 | 37 | 38 | 0.33 | 0.89 | 0.99996 |
|  | F7 | 69137 | 44 | 53 | 0.36 | 0.87 | 0.99984 |
|  | F8 | 73768 | 36 | 36 | 0.35 | 0.87 | 0.99997 |
|  | F9 | 71883 | 43 | 50 | 0.37 | 0.87 | 0.99990 |
|  | S1 | 72720 | 36 | 41 | 0.31 | 0.90 | 0.99992 |
|  | S2 | 71682 | 28 | 28 | 0.17 | 0.95 | 0.99997 |
|  | S3 | 70187 | 19 | 20 | 0.10 | 0.97 | 0.99997 |
|  | S4 | 73073 | 34 | 38 | 0.26 | 0.93 | 0.99990 |
|  | S5 | 71699 | 25 | 26 | 0.16 | 0.96 | 0.99996 |
|  | S6 | 69191 | 33 | 36 | 0.15 | 0.96 | 0.99993 |
|  | S7 | 65828 | 31 | 32 | 0.31 | 0.91 | 0.99995 |
|  | S8 | 69089 | 19 | 19 | 0.08 | 0.98 | 0.99999 |
|  | S9 | 69868 | 24 | 25 | 0.20 | 0.94 | 0.99996 |
|  | M1 | 58006 | 50 | 59 | 0.32 | 0.89 | 0.99979 |
|  | M2 | 60140 | 50 | 51 | 1.67 | 0.32 | 0.99997 |
|  | M3 | 53643 | 62 | 68 | 2.09 | 0.20 | 0.99993 |
|  | M4 | 39362 | 29 | 30 | 0.55 | 0.77 | 0.99990 |
|  | M5 | 51794 | 67 | 68 | 1.93 | 0.26 | 0.99996 |
|  | M6 | 50804 | 64 | 65 | 1.99 | 0.21 | 0.99996 |
|  | M7 | 58138 | 77 | 78 | 2.01 | 0.22 | 0.99995 |
|  | M8 | 50907 | 56 | 56 | 1.91 | 0.24 | 0.99996 |
|  | M9 | 49446 | 63 | 65 | 2.13 | 0.19 | 0.99992 |


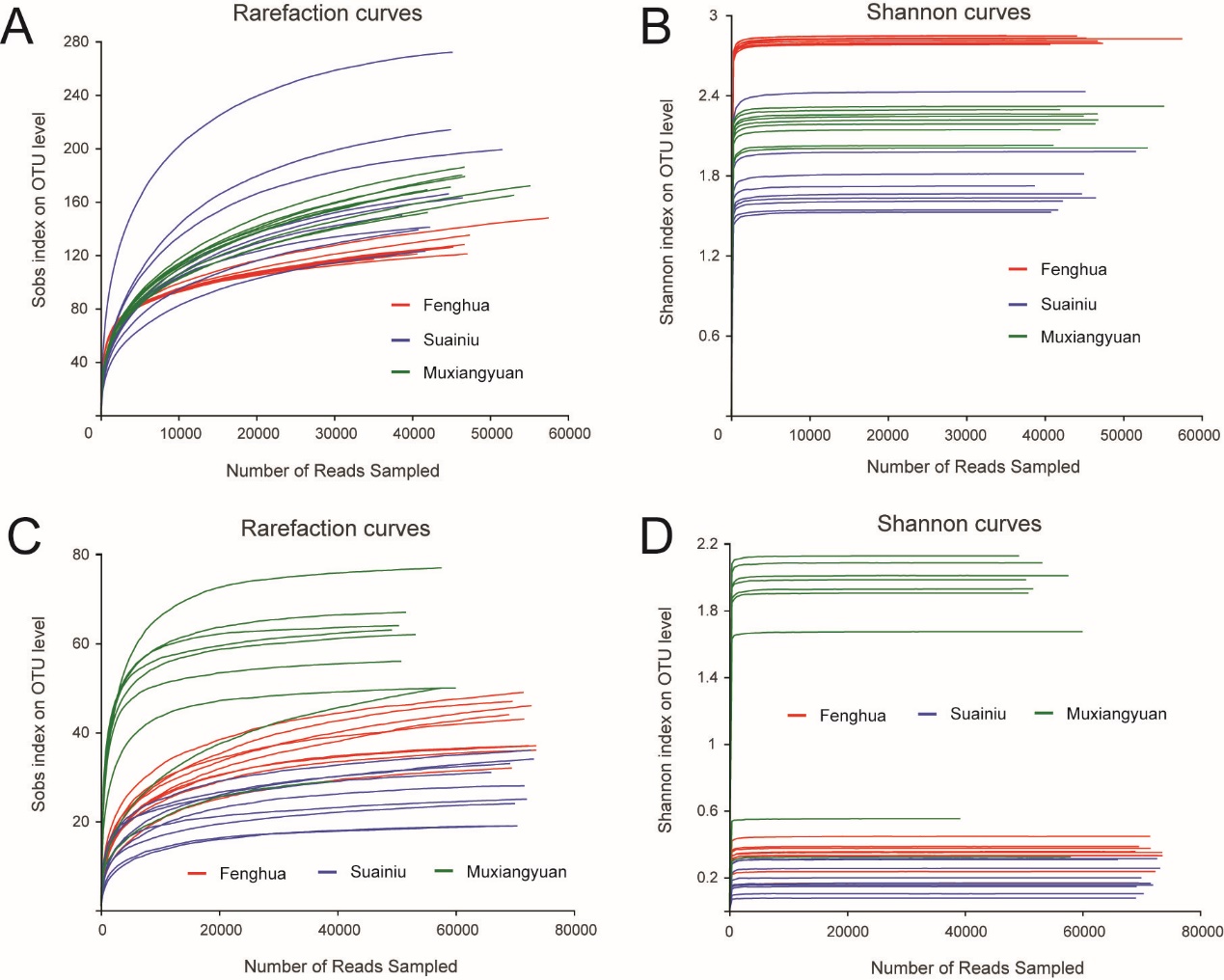


Supplemental Figure S2. Rarefaction curves (A and C) and Shannon diversity curves (B and D) for 16S rRNA (A and B) and ITS (C and D) sequencing analysis. OTU = operational taxonomic units.
